# The use of laser photobiomodulation as pre-anesthetic tissue management technique in reducing injection pain in children

**DOI:** 10.1186/s12903-024-04430-3

**Published:** 2024-06-22

**Authors:** Aliaa Abdelsalam Hamouda, Laila M. El-Habashy, Amani Khalil

**Affiliations:** Department of Pediatric Dentistry and Dental Public Health, Faculty of Dentistry, Alexandria University, Champollion St, Azarita, Alexandria 21527 Egypt

**Keywords:** Laser photobiomodulation, Low level laser, Injection pain, Local anesthesia, Children, Topical anesthesia

## Abstract

**Background:**

One of the main goals for pediatric dentists is to offer a painless anesthesia experience. Laser photobiomodulation is among the suggested strategies to decrease injection pain. So, this study aimed to assess the impact of laser photobiomodulation on local anesthesia (LA) injection pain in children and its effect on the efficacy of LA during pulpotomy and SSC procedures.

**Methods:**

The research was carried out as a randomized controlled clinical trial with two parallel group design. It involved 64 cooperative healthy children, age range from 5 to 7 years, each having at least one maxillary molar indicated for pulpotomy. Children were randomly allocated to one of the two groups based on the pre-anesthetic tissue management technique used: test group received laser photobiomodulation, while control group received topical anesthetic gel. Pain during injection, pulpotomy, and SSC procedures was assessed using physiological measures (Heart Rate (HR)), subjective evaluation (modified Face-Pain‐Scale (FPS), and objective analysis (Sound‐Eye‐Motor scale (SEM)).

**Results:**

A total of 64 children with mean age 6.23 ± 0.78 participated in this research. The mean HR scores were significantly lower in the laser PBM group during buccal and palatal infiltration injections. The SEM mean scores were significantly lower in the laser PBM group during both injections. For the FPS scale, the number of children who recorded satisfaction during injection was significantly higher in laser PBM group. There was no statistically significant difference in mean HR as well as in SEM and FPS scores between the two groups during pulpotomy and SSC procedures. Comparisons between the two study groups were performed using independent samples t- and Mann-Whitney U tests. Significance was set at p value < 0.05.

**Conclusion:**

Laser photobiomodulation is a promising non-pharmacological pre-anesthetic tissue management technique in children that offered less painful injection compared to topical anesthetic gel without compromising the effectiveness of LA.

**Trial Registration:**

ClinicalTrials.gov Identifier: NCT05861154. Registered on 16/5/2023.

**Graphical Abstract:**

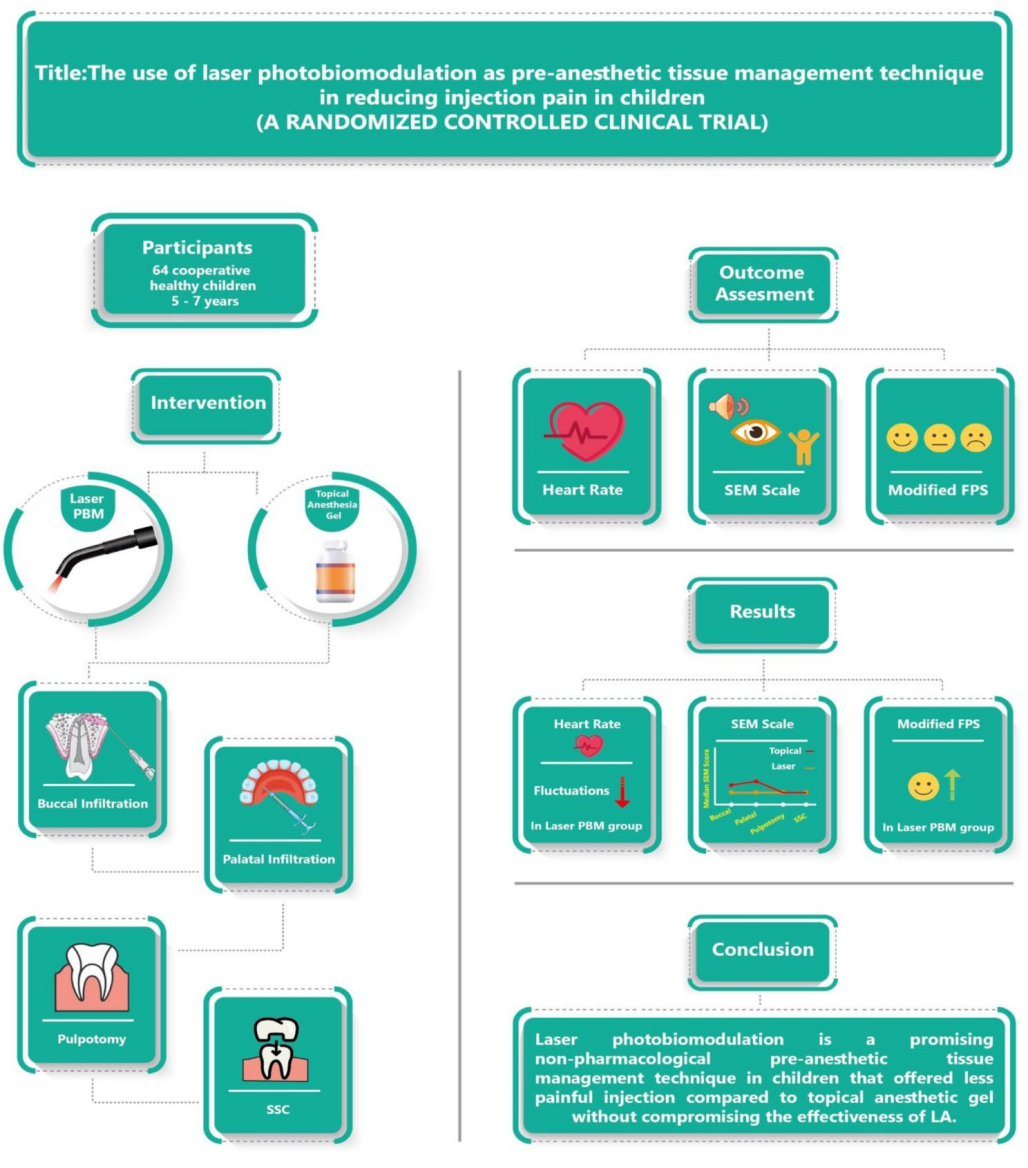

**Supplementary Information:**

The online version contains supplementary material available at 10.1186/s12903-024-04430-3.

## Background

In pediatric dentistry, local anesthesia (LA) injections are generally the most frightening and anxiety-inducing stimuli [1]. Several approaches have been used to reduce pain during the administration of the LA injection in children including: topical anesthetic agent, vibrating the tissue around the injection site during injection, pre-cooling the injection site, warming local anesthetic agent, buffering anesthesia solution, the use of electronic dental anesthesia, needle-free injection techniques, and computer-controlled injection systems. However, there is a lack of consensus on the effectiveness of these techniques since none of these strategies have completely relieved pain. Hence, there is always a need to develop novel efficient strategies to decrease injection pain.

In the last decade, several research have been conducted focusing on the use of lasers in dentistry practices. One of the suggested strategies in reducing injection pain is the photobiomodulation therapy (PBMT) being non-invasive, vibration-free, non-ablative and non-thermogenic intervention [2]. Laser photobiomodulation (PBM) is characterized by using parameters of low energy density, which is considered to be free of potential side effects [3]. Therefore, its effectiveness to alleviate the discomfort and reduce injection-related pain of LA in children would be of great benefit. It also has to be noted that photobiomodulation may’ increase the microcirculatory blood flow [4, 5].

The mechanism underpinning the alleviation of pain by PBMT is possibly highly complex. In spite of still not fully understanding the mechanism of its analgesic effect, the analgesic impact of PBM has been described in the literature and is employed in various clinical situations including the management of herpes [6], temporomandibular disorders [7], dentinal hypersensitivity [8], chronic facial myalgia [9], and postoperative pain following endodontic [10] or surgical procedures [11–13].

Photobiomodulation use in reducing injection-related pain has been tested in several studies [14–20]. However, the number of studies that targeted pediatric patients is limited [17–20]. And unfortunately, consistent results with these studies have not been achieved due to the variable clinical effects of varying laser parameters used and incomplete reporting of parameters.

Based on the data available from the literature, although laser PBM showed promising results in reducing pain in some studies, it’s obvious that there are not enough studies to make a definite conclusion regarding the effect of laser PBM on reducing injection pain, especially in pediatric populations. Therefore, this study was conducted to evaluate the effect of laser PBM as pre-anesthetic tissue management technique compared to topical anesthetic gel on reducing injection pain in children. We also aimed to evaluate its effect on LA efficacy during pulpotomy and stainless-steel crown (SSC) procedures for maxillary primary molars.

## Materials and methods

### Study design and setting

This study was conducted as a double blind randomized controlled clinical trial. This clinical trial involved 64 healthy children, with an age range between 5 and 7 years, who were attending the outpatient clinic of the Pediatric Dentistry and Dental Public Health Department. Children were assigned at random to two parallel groups according to the pre-anesthetic tissue management technique used with allocation ratio 1:1. The study took place at the laser technology clinic, Faculty of Dentistry, Alexandria University.

### Ethical consideration

The study was conducted after approval from the Research Ethics Committee, Faculty of Dentistry, Alexandria University (IRB No. 001056 –IORG 0008839) and according to the Declaration of Helsinki principle [21]. Verbal and written informed consent from parents or guardians of children and children’s assent were prerequisites for participation.

### Sample size estimation

The sample size was calculated based on a previous study [18] and considering 95% confidence level. The initial estimated sample size was 29 patients per group, which was adjusted to 32 to achieve an appropriate sample size. Consequently, the total required sample size was 64 patients (2 groups × 32 patients) [22]. The sample size was verified using MedCalc Statistical Software version 19.0.5 (MedCalc Software, Ostend, Belgium; https://www.medcalc.org; 2019).

#### Eligibility criteria

Children who met the following inclusion criteria were chosen to be enrolled in this study:

- Completion of the written informed consent form by parents/guardian.
- Children with an age range between 5 and 7 years old.
- Children with dental behavior score of 3 or 4 according to the Frankel behavior rating scale [23].
- Each child has at least one maxillary primary molar indicated for pulpotomy [24].
- Lack of history of allergy to the materials used for anesthesia and sulfite.
- Children free of any systemic disease or special health care needs.

#### Exclusion criteria

- Children who received any analgesic drugs at least 24 h before treatment.
- Presence of any inflammation or lesion at the injection site.
- Children who have previous bad experience of dentistry.
- Child presenting for emergency treatment of dental pain.

### Randomization and allocation concealment

Children were assigned at random using a computer-generated list to two parallel groups according to the pre-anesthetic tissue management technique used with allocation ratio 1:1. To ensure allocation concealment, every participating child received an exclusive serial number, employed during the randomization procedure. These numbers were written on identical sheets of paper accompanied by the name of the group to which each child has been assigned and enclosed within opaque envelopes bearing the child’s name [25]. The duty of storing the envelopes and unveiling them exclusively during the LA injection session was assigned to impartial personnel not involved in the trial, to ensure the concealment of the child’s assigned group from the outcome assessor.

### Blinding

In addition to the intervention received by both groups, the control group received a sham laser, and the test group received a sham gel to ensure that the participants were blinded. Also, the outcome assessor as well as the statistician were blinded.

### Clinical procedure

To ensure consistency, all clinical procedures were performed by the same pediatric dentist.

#### Preliminary screening visit

Children whose parents provided their permission to participate were examined and a full medical and dental history were taken.

No therapeutic interventions were performed at the first dental visit to foster a good relationship between the child and dentist and acquaint the child with dentistry [26]. Topical fluoride was applied to the child’s teeth, and both the child and their parents were given oral hygiene instructions.

#### Intervention visit

##### Pre-anesthesia tissue management

In the control group, a 20% Benzocaine topical anesthetic gel (Dharma Research, Inc. 5220 NW 72nd Ave Miami, FL 33,166 USA.) was applied. To increase the absorption of the topical anesthetic gel, soft tissues were dried with (2 × 2 cm) gauze. Topical anesthetic gel was applied at the site of needle penetration and was left in contact with the soft tissues for one minute to maximize its effect.

In the test group, a diode laser (Sirolaser blue laser system, Sirona Dental Systems GmbH, Fabrikstraße 31, 64,625 Bensheim, Germany) of Wavelength 660 nm was used and the laser parameters were set as follows: Power 0.1 W, energy 6 J, Continuous wave, energy Tip area 0.5 cm2, fluence 12 J/cm2 was applied at the site of needle penetration for 60 s. The laser was applied in contact mode at the sites of injection on buccal and palatal mucosa.

The device output power was checked using a power meter 3 times during the trial. Both the patient and the operator wore safety goggles during the radiation of laser.

Before each laser application, the output of the device was inspected using the aiming beam on a flat surface.

##### Local anesthesia injection

The procedure was videotaped to assess SEM scale.

##### Psychological child preparation

- The process of anesthesia administration was explained to all the children in simple terminology suitable for their age, using language free from any pain stimulating vocabulary.
- The child was placed in a supine position, aligning their head and chest parallel to the floor, while elevating their feet slightly. The operator’s hand was hiding the child’s sight field during LA injection, so the child never saw the needle.

Articaine hydrochloride 4% was injected with 1:100,000 epinephrine (ARTINIBSA, Inibsa Dental S.L.U, 08185 Lliçà de Vall, Barcelona, Spain) using a 27- gauge disposable short dental needle.

Standard technique for infiltration injection was performed on the buccal gingiva of the tooth in the approximate position of the root apex of the related tooth, where the mucosa of the cheek at the injection site was stretched and needle penetrated at a site between mucobuccal fold and mucogingival junction. 1.5 ml of the anesthetic solution was slowly deposited supraperiosteal. Buccal infiltration was then supplemented with palatal infiltration injection. Palatal injection was midway between gingival margin and mid palatine raphe, keeping the needle perpendicular to the palatal surface where approximately 0.2 ml of anesthetic solution was deposited [27].

Pulpotomy procedure was performed following the AAPD guidelines [24] followed by SSC preparation, selection, and cementation.

##### Outcome assessment

The following three approaches were used to assess the child’s reaction to pain:

###### A. Physiologic method (measuring HR)

The pulse oximeter was placed on the patient’s index finger and the patient was asked to remain still and avoid hand movements to ensure precision in the recorded readings. A baseline HR preceding the administration of LA was recorded, and subsequent measurements were conducted during the administration of LA, pulpotomy, and SSC restoration procedures, with heart rate recordings obtained at 2-minute intervals. The mean HR measurements were subsequently computed for analysis.

###### B. Objective method

The Sound, Eye, Motor (SEM) Scale (Table 1) was used to assess pain objectively. Each sound, eye, and body movement were rated on a scale of 1 to 4. The pain was then quantified by summing the score of these three outcomes and getting their mean during LA administration as well as during pulpotomy and SSC restoration procedures.

###### C. Subjective method

**Fig. 1:**
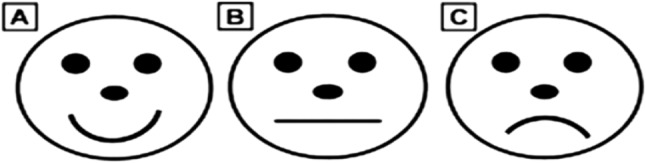
Modified face pain scale (FPS) **A**. satisfaction **B**. indifferent **C**. dissatisfaction

The modified Face Pain Scale (FPS) by Maunuksela et al. [28] (Fig. 1) was adopted which is comprised of three faces with various expressions denoting (from left to right): (a) satisfaction; (b) indifference; and (c) dissatisfaction. The patients were instructed to use this scale to report how they felt immediately following the LA injection and after pulpotomy and SSC procedures.

**Table 1:** SEM scale for objective assessment of pain

Parameter	Comfort	Mild discomfort	Moderate discomfort	Severe discomfort
Grade	1	2	3	4
Sound	No sound	Non-specific sound (probable pain)	Verbal complaint, louder sound	Verbal complaint shouting, crying
Eye	No sign	Dilated eye without tears (anxiety sign)	Tears, sudden eye movements	Crying, tears all over the face
Motor	Relaxed body and hand status	Muscular contraction, contraction of hands	Sudden body and hand movements	Hand movements for defense, turning the head to the opposite side.

### Inter and intra-examiner reliability

SEM scale was evaluated by the operator and by a blind impartial observer using the recorded video tapes. The exercise of measuring SEM based on videotapes was repeated after a 7-day break to obtain an appropriate level of examiner reliability. Intraclass correlation (ICC) was used to assess intra- as well as inter- examiner reliability, which ranged between 0.87 and 0.95 indicating excellent reliability between examiners and across time.

### Statistical analysis

Data analysis was conducted using IBM SPSS for Windows (Version 26.0). All variables were checked for normality. Comparisons of quantitative normally distributed variables between the two study groups were done using independent samples t-test, while comparison of qualitative ordinal variables and non-normally distributed variables was done using the Mann-Whitney U test. Comparing qualitative nominal variables between the two study groups was done using Chi-square test. As for within group, repeated measures ANOVA was used for quantitative normally distributed variables, and Friedman test for non-normally distributed and qualitative ordinal variables, and Cochran Q test for comparing qualitative nominal variables. Multiple pairwise comparisons using the Bonferroni adjusted significance level were performed after each of these tests. P value < 0.05 was used to determine significance.

## Results

**Fig. 2:**
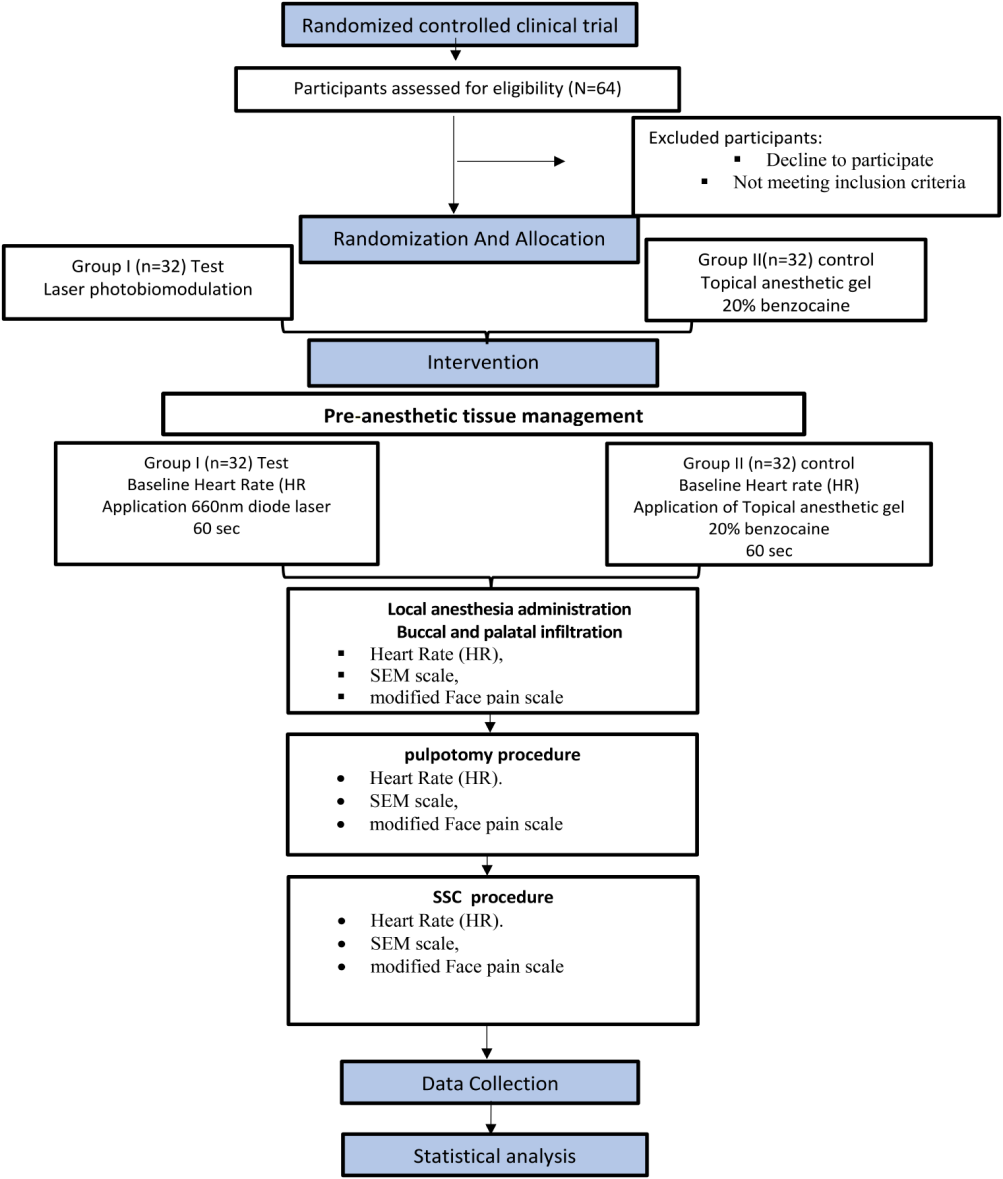
CONSORT Flow Chart Study

The CONSORT [29] checklist served as the protocol for reporting this trial (Fig. 2). A total of 64 children participated in the study 46.9% (*N* = 30) males, 53.1% (*N* = 34) females with a mean age of 6.23 ± 0.78. No significant differences were recorded between the two groups regarding age (*p* = 0.71), gender (*p* = 0.62) or tooth location (*p* = 0.21).

### Results of pain assessment based on physiological measure (HR) (Table 2)

There was a significant increase in mean HR during palatal injection compared to mean baseline HR in both groups with values for the test group (*p* = 0.01) and (P = < 0.001) for the control group (Appendix I).

**Table 2:** Comparison of heart rate (HR) between the two study groups across time

	Laser photobiomodulation	Topical anesthetic gel	Mean difference	95% CI	*P* value 1
	Mean (SD)				
Basal	103.00 (9.11)^a^	106.91 (12.51)^a^	-3.91	-9.39, 1.57	0.16
Buccal	104.53 (10.29)^ab^	113.78 (13.67)^b^	-9.25	-15.30, -3.21	0.003*
Palatal	105.97 (10.34)^b^	116.81 (13.87)^c^	-10.84	-16.96, -4.73	0.001*
Access opening	104.88 (8.68)^ab^	107.78 (12.73)^ab^	-2.91	-8.37, 2.55	0.29
SSC	103.59 (8.28)^ab^b	108.25 (12.14)^ab^	-4.66	-9.86, 0.55	0.08
P value 2	0.005*	< 0.001*			

P value 1: independent samples t-test was used

P value 2: repeated measures ANOVA was used

*statistically significant at p value < 0.05

a-d: different letters denote statistically significant differences between time points within each group – using Bonferroni adjusted significance level

As well, there was a significant increase in mean HR during buccal infiltration compared to mean baseline HR in the control group (p = < 0.001). while no statistically significant difference was detected between mean HR scores during buccal infiltration and base line in the test group.

Comparison of mean HR between the two study groups across time showed that the mean basal HR wasn’t significant between the two groups (*p* = 0.16). However, the mean HR during buccal and palatal injection was significantly higher in the control group compared to the test group with values (*p* = 0.003), (*p* = 0.001) respectively. While differences in mean HR between both groups didn’t reach the level of significance during pulpotomy (*p* = 0.29) and SSC (*p* = 0.08) procedures.

### Results of pain assessment based on objective measure (SEM scale) (Table 3)

When assessing the SEM mean scores from injection to SSC procedure for each group: in the control group the SEM mean scores were statistically significantly higher during buccal infiltration compared to pulpotomy (*p* = 0.02) and SSC (*p* = 0.01) as well as the SEM mean scores during palatal infiltration were statistically significantly higher compared to pulpotomy (p = < 0.001) and SSC procedures (p = < 0.001).(Appendix I) However, in the test group the SEM mean scores during palatal injection were statistically significantly higher than pulpotomy (*p* = 0.04) and SSC procedures (*p* = 0.03) (Appendix I). However, there was no statistically significant difference between the SEM mean scores during buccal infiltration compared to pulpotomy and SSC procedures.

**Table 3:** Comparison of SEM scores between the two study groups across time

		Laser photobiomodulation	Topical anesthetic gel	*P* value 1
Buccal	Mean (SD)	1.10 (0.21)^ab^	1.20 (0.22)^a^	0.04*
	Median (IQR)	1.0 (1.0, 1.0)	1.15 (1.0, 1.30)	
Palatal	Mean (SD)	1.16 (0.23)^b^	1.34 (0.29)^a^	0.006*
	Median (IQR)	1.0 (1.0, 1.30)	1.30 (1.0, 1.60)	
Pulpotomy	Mean (SD)	1.02 (0.07)^ab^	1.01 (0.05)^b^	0.56
	Median (IQR)	1.0 (1.0, 1.0)	1.0 (1.0, 1.0)	
SSC	Mean (SD)	1.01 (0.05)^a^	1.00 (0.00)^b^	0.32
	Median (IQR)	1.0 (1.0, 1.0)	1.0 (1.0, 1.0)	
P value 2		< 0.001*	< 0.001*	

P value 1: Mann-Whiteny U test was used

P value 2: Friedman test was used

*statistically significant at p value < 0.05

a, b: different letters denote statistically significant differences between time points within each group – using Bonferroni adjusted significance level

**Table 4:** Comparison of FACES scale between the two study groups across time

		Laser photobiomodulation	Topical anesthetic gel	*P* value
		*N* (%)		
Anesthesia	Satisfaction	25 (78.1%)	17 (53.1%)^a^	0.03*
	Indifferent	4 (12.5%)	6 (18.8%)	
	Dissatisfaction	3 (9.4%)	9 (28.1%)	
Pulpotomy	Satisfaction	26 (81.3%)	30 (93.8%)^b^	0.12
	Indifferent	4 (12.5%)	2 (6.3%)	
	Dissatisfaction	2 (6.3%)	0 (0%)	
SSC	Satisfaction	26 (81.3%)	30 (93.8%) b	0.13
	Indifferent	5 (15.6%)	2 (6.3%)	
	Dissatisfaction	1 (3.1%)	0 (0%)	
P value 2		0.84	< 0.001*	

P value 1: Mann-Whiteny U test was used

P value 2: Friedman test was used

*statistically significant at p value < 0.05

a, b: different letters denote statistically significant differences between time points within each group – using Bonferroni adjusted significance level

The SEM mean score in the control group during buccal injection was 1.20 ± 0.22 and during palatal injection was 1.34 ± 0.29, which were higher than the test group scores (1.10 ± 0.21) and 1.16 ± 0.23) respectively. The SEM scales’ findings revealed statistically significant lower scores during buccal infiltration injection as well as palatal infiltration injection in the test group compared to the control group with (*p* = 0.04) and (*p* = 0.006) respectively. However, there was no statistically significant difference in the SEM mean scores between the two groups during pulpotomy and SSC procedures, *p* = 0.56 and *p* = 0.32 respectively.

### Results of pain assessment based on subjective measure (modified FPS) (Table 4)

In the control group, the number of children who reported satisfaction after pulpotomy and SSC procedures was significantly higher than the number of children who reported satisfaction after anesthesia. In contrast, there was no significant difference detected when comparing the number of children who reported satisfaction after anesthesia and after the end of the dental procedures in the test group (Appendix I).

The analysis of FPS data between both groups revealed a significant difference in the patient’s satisfaction following injection (*p* = 0.03). Where, 78.1% of participants in the test group reported a satisfying experience following injection, and only three children reported a negative experience. In the control group, 53.1% satisfied patients were reported. Upon comparing FPS scores between the two groups during pulpotomy and SSC procedures there was no significant difference noted regarding satisfaction.

## Discussion

Reducing injection pain and increasing anesthesia efficiency are crucial for a successful treatment for pediatric dental patients, being critical in reducing anxiety and having influence on pain perception throughout different treatment procedures [27].

Laser photobiomodulation has shown its effectiveness in reducing injection pain in some previous studies [15–19], however, there is a very limited number of studies that addressed this topic in children and each study adopted a different protocol regarding the method of application, the laser type, mode, parameters used, sample chosen, and anatomical site tested. Furthermore, previous studies on children included children six years and above who were more capable of controlling their feelings and response to pain. Along with this data from the published literature, came our motivation to conduct this study to shed more light and add more information to this gap in the literature, where this study aimed to evaluate the effect of laser PBM as pre-anesthetic tissue management technique compared to topical anesthetic gel on reducing injection pain targeting children aged between 5 and 7 years to cover a wider range of children preschoolers (5–6) and schoolers (6–7) due to the different emotional, cognitive, and social development at this stage of life, and since PBM could modifies microcirculation at the injection site [4, 5] so we also aimed to test its effect on local anesthesia efficacy during pulpotomy and SSC procedures.

This clinical study used a parallel design, keeping the two groups entirely apart to prevent the detrimental effects of one technique on the behavior of the children during subsequent visits.

Given the fact that palatal injection is the most painful [30, 31], palatal injections and maxillary buccal infiltrations were the focus of this investigation. In this study both upper primary molars were selected.

In this study we adopted a diode laser with a wavelength of 660 nm and an energy density of 12 J/cm^2^ for the test group. Since, PBM is mainly induced using diode lasers with a therapeutic window of wavelengths ranging between (630–940 nm) which leads to deeper tissue penetration [32]. And according to the systematic review and meta-analysis done by Cronshaw et al. targeting the PBM dose parameter in dentistry when applying PBM in contact mode with tissues the recommended dose for pain relief is between 10 and 30 J/cm2 [33]. In the control group, a 20% benzocaine gel was applied, being the most common and potent topical anesthetic used in pediatric dentistry [34].

Given that the effectiveness of laser PBM in alleviating pain is still being studied, some research tested the paired effect of both topical anesthetic gel and laser PBM [18, 19]; while other trials studied only the effect of laser PBM exclusively [17, 20]. This has rendered the results to be inconsistent, where topical anesthetic gel application may have camouflaged PBM’s genuine effectiveness. Therefore, in this study laser PBM was applied alone as a pre-anesthetic tissue management technique this helped us to test its genuine effect.

The dental procedures in this study confined to pulpotomy and SSC procedures, being one of the most common pediatric dental treatments and one of the longest procedures so this helped us to test the effect of the intervention on the efficacy of LA.

It’s truly challenging to achieve accurate pain measurements chiefly when dealing with children, owing to their limited vocabulary, limited experiences, lower cognitive skills, and limited ability to express themselves [35]. Children’s self-reports of pain are questionable, because they are significantly affected by developmental, environmental, anxiety and psychological factors. So, in addition to self-report measures, objective and/or physiological measures should be addressed [35]. Accordingly, in this clinical trial we used the three methods of measuring pain: physiologically by monitoring the heart rate (HR) being a reliable physiological indicator of pain [36] and it eliminates any potential bias that could result from subjective and objective reporting of pain, secondly, objectively assessing pain using SEM scale, being used in several prior research and has shown its accuracy in measuring children’s pain [37, 38]. And finally, the modified face pain scale FPS from El Maunuksela et al. [28] was adopted for subjective pain measurement, which shows only three faces (satisfaction, indifferent, dissatisfaction) unlike the conventional FPS which comprise six faces, trying to make it easier to comprehend, interpret and to reduce the child’s confusion.

Our results revealed that laser PBM significantly decreased the injection pain, this was in agreement with Shekarchi et al. [17] who tested the effect of laser PBM (using 808 nm diode laser, with 250 mW power and 32.5 J/cm^2^ fluence in contact mode for 65 s) compared to topical anesthetic gel. The less pain experience in the laser PBM group could be contributed to the analgesic effect of laser PBM, where PBM alters nociceptive signals, decreasing the impulse transmission and firing frequency of the peripheral nerves [39]. PBM as well suppresses the conduction of type A and C pain fibers and hinders neurogenic inflammation [40]. Furthermore, PBM enhances β-endorphins release and decreases production of prostaglandins [39–41].

On the contrary, our results weren’t in accordance with Uçar et al. [18] who compared topical anesthetic gel application solely to topical anesthetic gel application coupled with PBM using 810 nm diode laser with power 0.3 W and fluence 69 J/cm^2^ for 20 s in non-contact mode. They found no statistically significant difference between the two groups based on the objective measures, although there were higher ‘no pain scores’ in topical anesthesia coupled with PBM group. Additionally, EL Bay et al. [19] who tested the effect of different PBM parameters using 940 nm diode laser in non-contact mode (with fluence 69 J/cm2, 103 J/cm2 and 138 J/cm^2^), they reported that there was no statistically significant difference in pain experience between the groups that received PBM coupled with topical anesthesia and the group that received placebo PBM with topical anesthesia. This could be attributed to different impact of different laser parameters implemented by each study on the tissue.

This study results showed no statistically significant difference in HR, FPS and SEM scales between the two groups during pulpotomy and SSC procedures, suggesting that PBM didn’t affect the LA efficacy, where anesthesia performed equally effective in both groups.

This is consistent with Shekarchi et al. [17] who claims that PBM application prior to LA had no influence on duration of LA and its effectiveness. Similarly, Ucar et al. [18] reported that using PBM coupled with topical anesthetic gel before LA injection had no influence on the duration or the effectiveness of the anesthesia given to the children.

This could be attributed to the nature of the sample chosen where all included children were cooperative. This helped to perform the procedure in a short time not more than 30 min, which didn’t exceed half the wash out time of articaine which is approximately 120 min [42]. This could be considered as a limitation of this study. So, future studies should consider uncooperative children.

Other limitations of this clinical trial are that it only tested a single set of laser parameters and only one anatomic site. Future research should test the impact of different laser parameters on pain of LA injection in pediatric dental patients at different anatomic areas during different dental procedures, which will furthermore verify our study’s findings and could contribute to establish guidelines or protocols for management with laser PBM in pediatric dentistry.

## Conclusion

According to our findings, we can conclude that laser photobiomodulation as pre-anesthetic tissue management strategy in children showed to be a promising non-pharmacological technique providing less painful injection compared to topical anesthetic gel without having an effect on LA efficacy. This can open a new era in reducing pain during LA injection and help in gaining child trust, assuring a favorable child behavior and cooperation during subsequent dental treatment procedures.

### Electronic supplementary material

Below is the link to the electronic supplementary material.

Supplementary Material 1

## Data Availability

The study’s dataset is accessible through the corresponding author upon a reasonable request, but it is not publicly accessible due to restrictions. This is because it includes information that might jeopardize the privacy of the research participants.

## Abbreviations

LA: Local Anesthesia
PBMT: Photobiomodulation Therapy
SSC: Stainless Steel Crown
CONSORT: Consolidated Standards of Reporting Trials Statement
ASA: American Society of Anesthesiologists
AAPD: American Academy of Pediatric Dentistry
HR: Heart Rate
SEM: Sound, Eye, Motor Scale
FPS: Face Pain scale
LLLT: Low Level Laser Therapy
