# Supplementary material for: The use of laser photobiomodulation as pre-anesthetic tissue management technique in reducing injection pain in children
Source: BMC Oral Health. 2024 Jun 22;24:717. doi: 10.1186/s12903-024-04430-3 (PMC11193196; doi:10.1186/s12903-024-04430-3)
Supplement: Supplementary file 1 — Supplementary Material 1 [file 12903_2024_4430_MOESM1_ESM.docx]

**Appendix I**

**Table 5 Post-hoc comparisons of heart rate (HR) at different timepoints within the two study groups**

| **Timepoint** | **Compared to** | **Laser**  **photobiomodulation** | **Topical anesthetic gel** |
| --- | --- | --- | --- |
|  |  | **P value** | |
| **Basal** | **Buccal** | 0.38 | **<0.001*** |
|  | **Palatal** | **0.01*** | **<0.001*** |
|  | **Access opening** | 0.21 | 1.00 |
|  | **SSC** | 1.00 | 0.06 |
| **Buccal** | **Palatal** | 0.06 | **<0.001*** |
|  | **Access opening** | 1.00 | **<0.001*** |
|  | **SSC** | 1.00 | **<0.001*** |
| **Palatal** | **Access opening** | 1.00 | **<0.001*** |
|  | **SSC** | 0.20 | **<0.001*** |
| **Access opening** | **SSC** | 0.06 | 1.00 |

*Statistically significant using Bonferroni adjusted significance level

**Table 6 Post-hoc comparisons of SEM at different timepoints within the two study groups**

| **Timepoint** | **Compared to** | **Laser**  **photobiomodulation** | **Topical anesthetic gel** |
| --- | --- | --- | --- |
|  |  | **P value** | |
| **Buccal** | **Palatal** | 1.00 | 0.73 |
|  | **Pulpotomy** | 0.33 | **0.02*** |
|  | **SSC** | 0.25 | **0.01*** |
| **Palatal** | **Pulpotomy** | **0.04** | **<0.001*** |
|  | **SSC** | **0.03** | **<0.001*** |
| **Pulpotomy** | **SSC** | 1.00 | 1.00 |

*statistically significant using Bonferroni adjusted significance level

**Table 7 Post-hoc comparisons of FACES scale at different timepoints within the two study groups**

| **Timepoint** | **Compared to** | **Laser**  **photobiomodulation** | **Topical anesthetic gel** |
| --- | --- | --- | --- |
|  |  | **P value** | |
| **Anesthesia** | **Pulpotomy** | 1.00 | **0.005*** |
|  | **SSC** | 1.00 | **0.005*** |
| **Pulpotomy** | **SSC** | 1.00 | 1.00 |

*statistically significant using Bonferroni adjusted significance level
